# Implementation of an ICU follow-up clinic: outcomes and patient satisfaction after 1 year

**DOI:** 10.1186/cc12476

**Published:** 2013-03-19

**Authors:** G De la Cerda

**Affiliations:** 1 Queens Hospital, London, UK

## Introduction

The aim was to analyse the outcomes and patient satisfaction of a recently implemented ICU follow-up clinic. These clinics are National Institute for Clinical Excellence recommended [1].

## Methods

A retrospective analysis of prospective collected data from January to December 2012. The clinic is run monthly by an ICU consultant and a critical care outreach sister. Criteria to be invited to the clinic are mechanical ventilation ≥3 days. Patients filled an anonymous satisfaction survey after the clinic.

## Results

**Figure 1:**
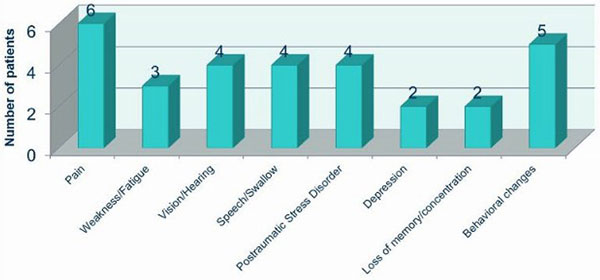
**Physical and nonphysical morbidity**.

**Figure 2:**
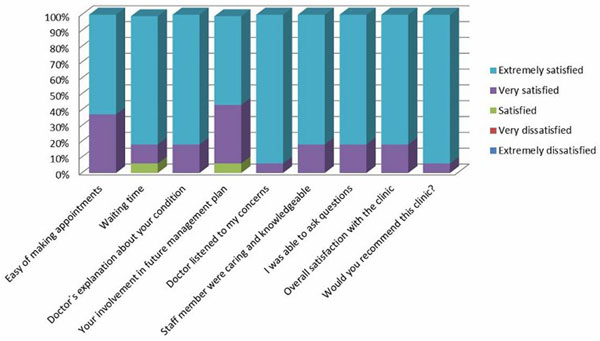
**Patient satisfaction survey**.

Our attendance rate is 50% (26 patients), which is similar to other series reported in the literature. Those patients who attended the clinic required a longer length of mechanical ventilation (5.3 days vs. 7.1) and a longer length of stay in the ICU (7.6 vs. 13) and in hospital (14 vs. 28). We identified a wide range of physical and nonphysical morbidities on these patients (Figure 1). We referred them to the appropriate specialities. Patients were very satisfied with this new service (Figure 2).

## Conclusion

Our follow-up clinic has enabled us to identify a wide range of complications related to ICU admission and coordinate their future management. This clinic improved patient satisfaction.
